# A qualitative exploration of parental perspectives on quality of care for children with serious illnesses

**DOI:** 10.3389/fped.2023.1167757

**Published:** 2023-07-28

**Authors:** Felicia Jia Ler Ang, Cristelle Chu-Tian Chow, Poh Heng Chong, Teresa Shu Zhen Tan, Zubair Amin, Siti Nur Hanim Buang, Eric A. Finkelstein

**Affiliations:** ^1^ Lien Centre for Palliative Care, Programme in Health Services and Systems Research, Duke-NUS Medical School, Singapore, Singapore; ^2^ Department of Paediatrics, KK Women’s & Children’s Hospital, Singapore, Singapore; ^3^ HCA Hospice Limited, Singapore, Singapore; ^4^ Department of Paediatrics, Khoo Teck Puat-National University Children’s Medical Institute, National University Hospital, Singapore, Singapore; ^5^ Department of Neonatology, Khoo Teck Puat-National University Children’s Medical Institute, National University Hospital, Singapore, Singapore; ^6^ Department of Paediatrics, Yong Loo Lin School of Medicine, National University of Singapore, Singapore, Singapore; ^7^ Global Health Institute, Duke University, Durham, NC, United States

**Keywords:** process assessment, quality of care, grounded theory, quality indicators, patient experience, parents, pediatrics, palliative care

## Abstract

**Introduction:**

Being responsive to end-users is essential to good care. Limited in-depth exploration of parental perspectives on care received by children over the course of serious illness has hindered the development of process measures to evaluate quality of care. Our objective was to identify the key process indicators prioritized by parents in the care of seriously ill young children and develop a framework to guide assessment of quality of care.

**Methods:**

This qualitative study followed Charmaz's Constructivist Grounded Theory. In-depth semi-structured interviews were conducted with parents of young children with serious illness in Singapore. Participants were sampled across various healthcare settings, children's ages, and illness categories. Theoretical sampling and constant comparative analysis were used to generate initial, focused, and theoretical codes, which informed construction of a conceptual framework.

**Results:**

31 parents participated from July 2021 to February 2022. Initial and focused coding generated 64 quality of care indicators describing key care practices, interactions, and procedures. Indicators were categorized under four themes: (1) efficient healthcare structures and standards, (2) professional qualities of healthcare workers, 3. supporting parent-caregivers, and 4. collaborative and holistic care. Theoretical coding led to the development of the “PaRental perspectives on qualIty of care for Children with sErious iLlnESSes (PRICELESS)” framework which summarizes elements contributing to the parental perception of quality of care.

**Discussion:**

The identified process indicators will facilitate the development of standardised parent-reported measures for assessing service quality and benchmarking among providers. The framework provides overall guidance for conceiving quality improvement initiatives.

## 1. Introduction

Each year, over 21 million children suffer from serious illnesses [defined by Together for Short Lives (1, 2)], impacting their daily functioning and quality of life (3, 4). While new treatments have improved survival rates (5), they have also exacerbated caregiver burden (6). End-user experience is a core outcome in healthcare delivery (7) because it is linked to resource utilization, clinical effectiveness, and safety (8). Family-centred care recognizes the child and parent as a unit (9) and acknowledges the vital role parents play in their child's care (10). Thus, parents serve as critical sources of experiential information as they navigate the healthcare system on behalf of their child (11), making them essential end-users even if they are not patients. Moreover, there is equivocal current evidence of reliability and validity of patient-reported measures when used in very young children below 8 years (12). Insight into parental end-user priorities is thus crucial when considering the quality of care for seriously ill children (12), especially since parents themselves are at risk of adverse outcomes (13).

Donabedian's framework of healthcare quality emphasizes that care processes connect input (“structures” of care) with output (“outcomes” of care) (14). Process quality indicators refer to well-defined indicators describing how care practices, interactions, or procedures take place (15). While previous research has defined broad themes such as the Patient- and Family-Centered Care approaches (16), there remains limited insight into the specific care processes parents prioritize. Furthermore, limited research has explored parental perspectives on quality of care for seriously ill children across care settings (17, 18). This leaves a gap in understanding the processes that parents prioritize from various providers (e.g., hospital vs. community-based care), or across healthcare workers (e.g., doctors, allied health professionals, medical social workers, nurses), all of whom are part of the family's care network. Given that previous research has primarily focused on single care settings (19–21), there exists a knowledge gap limiting the development of comprehensive yet meaningful measures for service assessment across the care continuum (22). For these reasons, recent work has emphasized the importance of enhancing care frameworks that can cross age groups, conditions, and care settings (23).

Therefore, we undertook this study to (1) determine key process indicators of quality of care for seriously ill children from the parental perspective, and (2) develop a consolidated framework to guide quality measurement and improvement initiatives. Findings should apply across care settings and throughout the illness trajectory.

## 2. Materials and methods

We structured this report according to the Consolidated Criteria for Reporting Qualitative research (COREQ) checklist (Supplementary File S1). Given a dearth of prior research into parental end-user perspectives on quality of care relevant to the range of pediatric serious illnesses and care settings, we adopted Charmaz's Constructivist Grounded Theory to guide data collection and analysis. This methodology is particularly suitable as it aims to produce an explanatory theory through inductive analysis, uncovering a process underpinning the area of inquiry (24, 25).

### 2.1. Participants

Eligible participants were adult (≥21 years) parents of young children (<8 years old) diagnosed with serious illness(es) in Singapore, an ethnically and socio-culturally diverse country in Southeast Asia. Since needs and trajectories can differ by age (26), purposive sampling was adopted for maximum variation in terms of children's age groups (<1, 1 to <3, 3 to <5, 5 to <8 years old) and serious illness categories. Parents were also purposively recruited across service delivery settings (e.g., home, hospice, community-based organizations, inpatient care, intensive care units, and specialized outpatient clinics) (1). This approach distinguishes our study from previous research, which often focused on specific sites, enabling a more holistic understanding of the illness journey. As emergent codes and themes arose, theoretical sampling was performed subsequently—parents with children at varying points of the illness trajectory were recruited to explore if their perspectives differed.

### 2.2. Procedures

Participants were either referred by partnering healthcare workers (HCW) to the first author or recruited via social media platforms of collaborating organizations. A semi-structured interview guide (Supplementary File S2) was used. It was piloted among two experts in pediatrics and palliative care who reviewed and improved several iterations of the guide for understandability and acceptability. To sensitize participants to the study topic, the interview guide began by explaining that researchers aimed to understand and ensure the delivery of quality care by care providers for the child. It then explored the parents' perspectives on important processes, challenges, and facilitators in the child's daily life, key services and behaviors from HCW, and gaps in current care. It also delved into the parents' own experiences, priorities, and expectations in relation to their child's healthcare needs. Field notes were taken together with relevant contextual information. All interviews were audio-recorded and transcribed verbatim for analysis. Consistent with Charmaz's methodology, the interview guide was updated as analysis progressed to foster deeper exploration of emerging concepts. For example, later interviews intentionally explored parents' perceptions on the relationships between various concepts that had surfaced during ongoing data analysis. This included asking parents how they perceived emerging categories were related, or whether they perceived any priorities had changed over time.

### 2.3. Data analysis

The constant comparative method was applied throughout to generate data and theory (27). A team of four female coders (CC, TT, SB and FA) familiarized themselves with the data by reading and re-reading coded transcripts. All transcripts were independently coded by two coders before discussion with the entire team. Initially, transcripts were coded line-by-line into numerous small segments (initial coding) annotated with detailed descriptions and excerpts. At focused coding, initial codes were reorganized along their properties and dimensions, thereby generating specific process indicators that represent parental perspectives on quality of care. Finally, the focused codes (process indicators) were abstracted through further refinement (theoretical coding), wherein we synthesized the interconnected concepts, relationships, and explanations from the data, ultimately culminating in the development of an overarching framework of quality of care. The process continued iteratively until the team concurred that data analysis had reached saturation, when all emerging themes were accounted for and additional data from successive interviews did not yield new insights (28).

### 2.4. Research team and reflexivity

The multidisciplinary coding team was composed of three leading clinicians experienced in caring for children with serious illnesses in their fields (CCTC, TSZT, SNHB) and a health services researcher with a background in psychology (FJLA). FJLA, who had no prior relationship with any participant, conducted all interviews. Throughout the process of analysis, the team reflected on how emerging findings might be influenced by their own biases. Coders served as peer debriefers by corroborating all analyses in group discussions and all ongoing data analysis and discussions were documented on a collaborative team document. Member-checking and expert validation were conducted by disseminating early findings to an expert panel of HCW caring for seriously ill children, participating parents, and partnering researchers to strengthen credibility and transferability of findings.

### 2.5. Ethical considerations

The Institutional Review Board (IRB) at the National University of Singapore approved the study (NUS-IRB-2021-362). Informed consent included permission to audio record interviews and use coded data to report qualitative findings. Participants were reimbursed SGD20 (approximately USD15).

## 3. Results

Thirty-two eligible parents were invited to participate in the study; 31 consented (94% response rate) and none dropped out. Parent and child characteristics are summarized in Table 1. Twenty-nine interviews were conducted via videoconference due to prevailing Covid-19 restrictions between July 2021 and February 2022. Three participants preferred to be interviewed in a group. All interviews were conducted in English only in the presence of the participant and interviewer, with each session lasting between 29 and 111 min (average: 57 min). No repeat interviews were required.

**Table 1:** Characteristics of participating parents and their children in the study (*n* = 31).

Parent characteristics [number (%)]		Child characteristics [number (%)]	
Female gender	23 (74%)	Female gender	18 (58%)
Mean age, years (SD)	37 (6)	Age group	
Ethnicity		0–<1	7 (23%)
Chinese	19 (61%)	1–<3	11 (35%)
Malay	7 (23%)	3–<5	7 (23%)
Indian	1 ((3%)	5–<8	6 (19%)
Others	4 (13%)	Category of diagnosed condition(s)^a^	
Married	27 (87%)	Category 1	6 (19%)
Religion		Category 2	6 (19%)
Christianity	6 (19%)	Category 3	6 (19%)
Buddhism	4 (13%)	Category 4	13 (42%)
Catholic	5 (16%)	Mean number of months since diagnosis (SD)	28 (21)
Free thinker	3 (10%)		
Taoism	3 (10%)		
Islam	10 (33%)		
Education			
Post-secondary level	27 (87%)		
Secondary school or ITE	4 (13%)		
Mean number hours spent on caregiving per week (SD)	85 (55)		
Caregiving roles (Answered “Yes”)			
Physically provide care to child (e.g., help with day-to-day activities)	29 (94%)		
Ensure provision of care (e.g., supervise helper to look after child)	24 (77%)		
Make decisions about treatments the child receives	30 (97%)		
Pay for the medical and health care expenses	27 (87%)		
Caregiving status			
Sole or primary caregiver	13 (52%)		
One of few caregivers	12 (48%)		
Employment			
Stopped working to take care of child	5 (16%)		
Full-time job	18 (58%)		
Homemaker	2 (6%)		
Unemployed	4 (13%)		
Others	2 (6%)		

^a^ Categorization as defined by Together for Short Lives (formerly known as the Association for Children's Palliative Care): Category 1. Life-threatening conditions for which curative treatment may be feasible but can fail; Category 2. Conditions where premature death is inevitable; Category 3. Progressive conditions without curative treatment options; Category 4. Irreversible but non-progressive conditions causing severe disability, leading to susceptibility to health.

**Figure 1:**
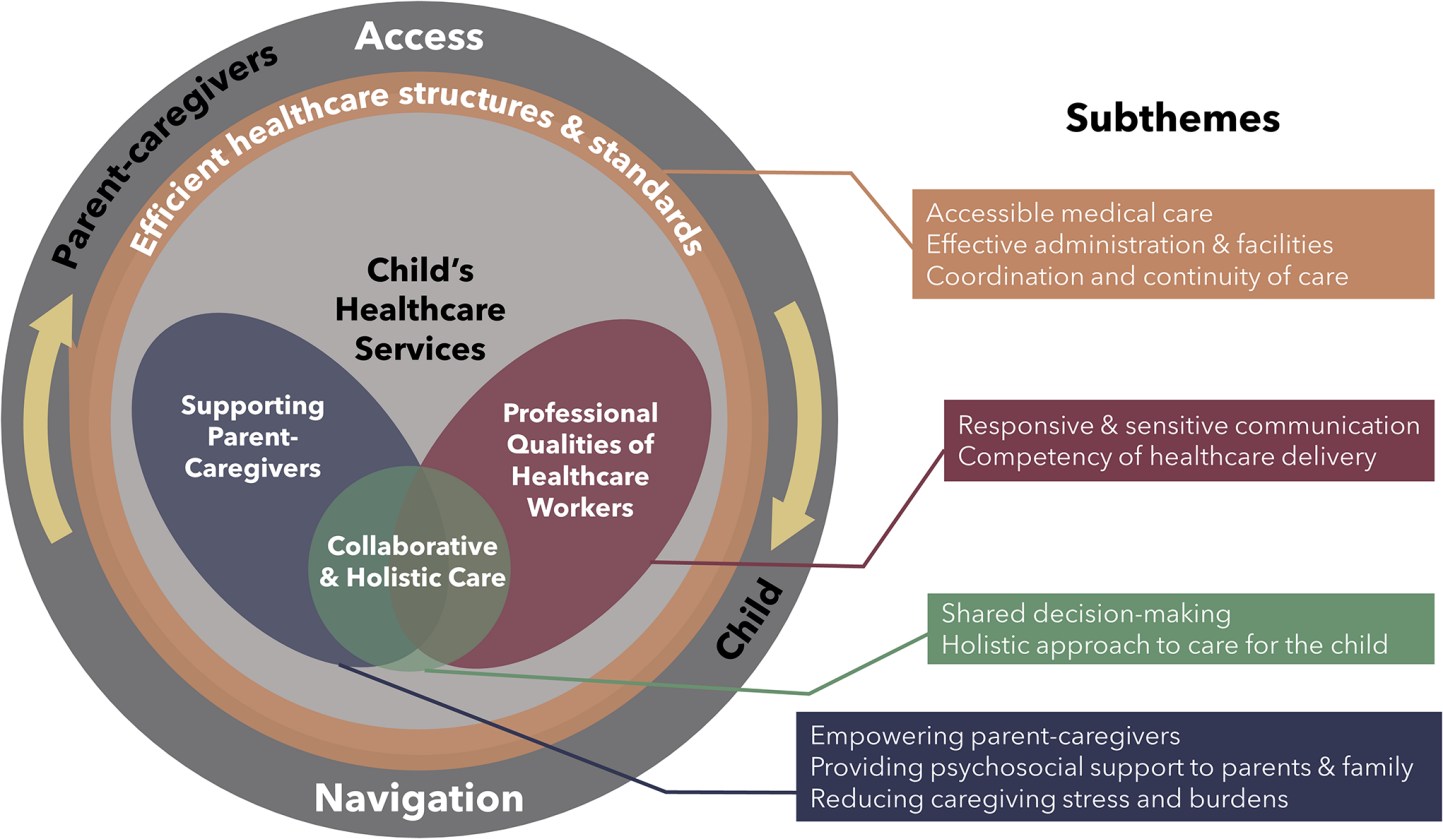
PRICELESS: PaRental perspectives on qualIty of care for children with sErious iLlnESSes theoretical framework.

Theoretical coding led to development of the PRICELESS framework: PaRental perspectives on qualIty of care for Children with sErious iLlnESSes (Figure 1). The proposed PRICELESS framework presents a holistic model consisting of an outer ring, which focuses on parent and child access to and navigation of healthcare services, and an inner circle, which pertains to the provision of care. The arrows depict the cyclical relationship between access and navigation. The importance and cyclical nature of accessing and navigating often complex care setups and services are captured in the following quote:

“[Whenever I am unsure what to do], I text the doctors and nurses and ask them what to do about it, they will tell me how to go about it. And then they will even try to go all the way to think of alternatives for me. […] That’s quite important, to know that when I need any advice or support, they’re always there […] So I have all the support that I need […] like now, I’m thinking of putting him to school, and I understand from [his doctor and several charitable organizations] that there [are] special school [options]… so there’s follow up. And I can always [reach out to them] if I'm concerned, which is what I've been doing… they will tell me [what I should do].”—PID27.

Initial and focused coding generated 64 process indicators of quality care from parental perspectives. From these indicators, we identified 10 subthemes that were then synthesized and summarized into four overarching themes: “professional qualities of HCW”, “collaborative and holistic care”, “supporting parent-caregivers”, and “efficient healthcare structures and standards”. Table 2 presents illustrative quotes for each subtheme, and Supplementary File S3 offers a full list of indicators, subthemes, and themes, with corresponding quotes.

**Table 2:** Four themes and ten subthemes encompassing quality of care captured in the PRICELESS: PaRental perspectives on qualIty of care for children with sErious iLlnESSes framework.

	Theme^a^	Subthemes and illustrative quotes
1	Professional Qualities of healthcare workers	*Theme 1*. Professional Qualities of healthcare workers: This represents parent's perception of the capabilities and capacities of their child's care team and workers, and should be considered a priority at the start of any illness journey. This observation arose from close examination of parents at differing points of the illness trajectory, influencing relationships with the care team downstream. 1.1 Responsive and sensitive communication: *“They like to push you to do, to decide on things, when [we wanted] things [to] be taken at a slower pace […] they should [not] push [us] to discharge [our] child [if we don’t feel ready to bring her home].”*—PID06. 1.2 Competency of healthcare delivery: *“when we arrive […] they won’t just brush it off like that, they will ask the doctor, like a specialist to come and take a look, make sure, [that is how] I can be assured that he is in good hands.”*—PID27.
2	Supporting parent-caregivers	*Theme 2*. Supporting parent-caregivers recognizes and thus alleviates the demands on parent-caregivers. 2.1 Empowering parent-caregivers: *“Give the caregivers a chance to voice out for their child. […] I am her voice.”*—PID06. 2.2 Providing psychosocial support to parents and family: *“I was heartbroken… because the doctors painted a picture of a future that is really, really bad. […] you are pushed to the corner where you have no other choice*…* I would have liked to speak to someone else.”*—PID07. 2.3 Reducing caregiving stress and burdens: *“Being the main caregiver is not easy [*…*] [With] this [respite care option], we are able to actually have self-care […] [before respite care], I was always on the edge, was always angry […] People didn’t understand. Why is it so important for you to rest? Is it [because] you don’t love your child? […] But I need to be strong, I need to be sane, to take care of my child!”*—PID13.
3	Collaborative and holistic care	*Theme 3*. Collaborative and holistic care reflects the value of a stable family-provider partnership in long-term delivery of family-centered care. 3.1 Shared decision-making: “*[After discussions], they will say [clicks tongue] yeah lah [expressive slang], you are the mummy, you know what’s comfortable. [*…*] there is a common understanding. I think that's important.”*—PID25 3.2 Holistic approach to care for the child: “*I can see from my child here, her mental [state], she's more traumatized [*…*] mental issue is also a big part [of their care]. I'm afraid they do not want to live anymore… [their mental wellbeing], it's also a big thing.” –PID13*
4	Efficient healthcare structures and standards	*Theme 4*. Efficient healthcare structures and standards represents the functional importance of having a robust structure for assessing quality of care. 4.1 Accessible medical care: *“[…] everyone that we try to ask for some advice or help… are very helpful […] whenever we try to call them [in any scenario] *…* They always help us, inform us, this is what you're going to do.”*—PID01. 4.2 Effective administration and facilities: *“They need to be more flexible with special needs child [in the hospital] […] the person in front [said], only one person can go in… but I need help!”*—PID09. 4.3 Coordination and continuity of care: *“…we were struggling [because] there wasn't like a so-called primary doctor, that coordinates everything, because [BF] has so many issues. So, she sees a lot of doctors and usually they just focus on their specialty […] having one overall doctor in charge, who really understands the case and understands the family needs [would have helped us a lot].”*—PID07

^a^ The ten subthemes and four themes accommodate all 64 quality of care process indicators.

“Efficient healthcare structures and standards”, situated as the intermediary link between the outer ring and inner circle of the framework, plays a crucial role as a mediator for facilitating access and navigation between parent-caregivers and HCW within the child's healthcare network.

The inner circle represents actual care delivered by HCW. “Collaborative and holistic care”, as an overarching ethos, guides the approach to healthcare delivery. This theme emphasizes an integrated and coordinated approach to care, while acknowledging the central role of both parents and HCW in caring for seriously ill children. Findings indicate that parents do not compartmentalize quality of care priorities across disciplines or providers. Instead, they perceive independent providers across various disciplines and health and social services as forming a cohesive care experience. The following quote captures this sentiment:

“The doctors and the nurses and the place itself […] they literally become friends and family, they literally become [my child]’s auntie… only after we get all of [the members of my child’s care team], the cardiologist, the ENT, all the consultants [onto the same page] … then we will move through that surgery […] other than the doctors and the nurses though, the cleaners also played a very important part. We love them… And our MSW [medical social worker] has played a very, very, very big part in bringing [my child] up… [she made things] possible … like now, early intervention [is] the only thing that we are actually looking forward to.”—PID18.

In contrast, “professional qualities of healthcare workers” and “supporting parent-caregivers” focus on the specific attributes of HCW and their actions towards parents and children. All four themes and 10 subthemes collaboratively determine overall quality of care.

The circular nature of the framework symbolizes the ongoing care journey undertaken by parents and HCW. It signifies that the experience of illness and care is cumulative, rather than being limited to discrete events, the essence of which can be captured in the following:

“It was a very long journey of [clinical investigations], tests and all, [to] narrow down [the condition] … the first two years of this journey [were] the most difficult. […] I’ve been [on] the journey [for several years] by now […] and I know, if I had compassionate doctors [and] a community that supported me at the very start of this journey, it would have been a lot more helpful.”—PID20.

### 3.1. Theme 1. Professional qualities of HCW

This theme captures the behaviors and attitudes that promote trust and confidence in HCW.

#### 3.1.1. Responsive and sensitive communication

Is woven throughout the data, reflecting the significant role that communication plays in defining healthcare experiences. Effective communication with families involves *communicating in a manner that is sensitive to parents' needs* and ensuring HCW *give parents time and space to make decisions without pressuring them* to minimize parental distress. Given that their child's illness(es) predisposes them to sudden clinical deterioration, HCW's should *avoid causing additional stress due to a lack of appropriate sense of urgency when communicating with parents,* since unanticipated communications can cause significant anxiety, for example:

“The doctor [woke] me up at around 2am. I thought that something happened to my child! And the only question she [wanted] to ask? Whether any of your family members [smoke]!”—PID20.

Responsive communication also involves effective information-sharing—respect of the parental right to information by ensuring HCW provide information on child's condition in a timely manner, while using understandable language and methods to communicate. The importance of these processes is captured here:

“They [did] not really update us about what [was] going on. […] On the week of discharge then we realized [wow], actually [my child has] so many [issues]? […] I don't know what I don't know! […] Now too late already then you tell me [sic]?”—PID26.

Given increasingly diverse ethnic and religious social contexts, HCW should *respect the spiritual or religious customs and beliefs of parents* to enable parents to tap into this source of strength during challenging times. Ultimately, parents value the relationships they develop with HCW over time and appreciate those who *make efforts to build parental trust in HCW* and *present themselves in an honest manner*.

#### 3.1.2. Competency of healthcare delivery

Begins with *responsiveness in managing the child’s medical issues.* This can be challenging to resolve as seriously ill children tend to have multiple concomitant distressing conditions. *Attending to the child within a reasonable amount of time,* particularly during unplanned hospitalizations where waiting times may be extended, is emphasized given seriously ill children are prone to clinical deterioration. Parents also experience significant distress if their child had unresolving symptoms or was experiencing excessive pain. Thus, they prioritize *providing symptom management to ensure child physical comfort* and *avoid unnecessary treatments and investigations* with the aim of maintaining the child's quality of life and limiting suffering:

“[sighs loudly] I had to keep on advocating for her, to stop giving her [certain drugs], she’s okay, she’s not dying from this [symptom] […] The nutritionist will want to up her feed until we can leave… It *has* to be a certain rate… they just want to do all this stuff […] they will want to put a drip in her. And she has very bad veins. So… they're basically popping, they’re basically, trying to find a new vein everyday and…. it’s quite traumatic for her.”—PID14.

Given the sense of powerlessness parents often feel, HCW who *reassure parents of their expertise in the field* and *take responsibility and accountability for child's wellbeing* can promote and build a trusting parent-provider relationship, especially when the child is admitted to healthcare facilities. A perceived breach of trust may be detrimental:

“I started staying very long hours with her after [my child was injured under their care] […] until now there wasn’t a concrete or an acceptable conclusion to this [incident] […] And my trust level went down to zero for that.”—PID31.

### 3.2. Theme 2. Supporting parent-caregivers

HCW play crucial roles in supporting parent-caregivers juggling between caring for their child's complex needs, for themselves, and the rest of the family.

#### 3.2.1. Empowering parent-caregivers

Revolves around supporting parents' role as medical-caregivers by equipping parents with skills to confidently deliver out-of-hospital care and providing anticipatory medical advice for parents to recognize when child's condition deteriorates. Equally important is providing parents with opportunities to bond with their child during admissions to healthcare facilities as this can help to maintain their parental role. It is also important to create an encouraging environment for parent-caregivers by acknowledging and affirming parents' efforts in caring for their child, for example:

“They always encouraged us […] “You’re good! You’re amazing!” […] Even though we [make mistakes] […] it’s really one of the highlights and I think that’s [kept] us going.”—PID18.

Given the intense and complex roles parents play in caring for seriously ill children, they value *provision of opportunities for caregivers to advocate or speak up for their child*:

“[…] you can always disagree, but… give the caregivers a chance to voice out for their child. Especially like my child, she is nonverbal […] I am her voice. If I don’t tell you that she deserves this, then who else is she, who else can she rely on?”—PID06.

Some parents desire *provision of opportunities for parents to give back to the special needs community**,*** such as by supporting other families, through which they derive a sense of purpose.

#### 3.2.2. Providing psychosocial support to parents and family

Involves *showing genuine care and concern* and *providing a compassionate listening ear*. These behaviors strengthen the parent-provider relationship and establish a sense of security that allows parents to relieve their emotions. HCW also need to regularly navigate a fine balance between *supporting parents' hopes for their child* while *preparing parents for what may lie ahead*, to manage parental despair while bolstering parents for potentialities:

“I was heartbroken… because the doctors painted a picture of a future that is really, really bad. […] [I felt] pushed to the corner where [I had] no other choice… You [just want] some hope that termination is not the only choice. I would have liked to speak to someone else.”—PID07.

Providing parents with emotional and physical space to grieve after delivering a serious diagnosis and towards the end-of-life care allows parents to process the news at their own pace. Attending to the psychosocial needs of the family unit resulting from the child's condition is repeatedly emphasized for siblings and other family members who may be struggling with understanding or coping with the child's illness. Finally, parents highlighted accessibility to parent support networks for informational and psychosocial support. Mutual parent-support is a powerful resource for parents, whereas a lack of access to such networks often heightens feelings of isolation:

“It was a struggle… to face everything alone [but if] you allow these parents and other parents who are facing similar conditions [to unite], you bring them together, it actually helps a lot.”—PID06.

#### 3.2.3. Reducing caregiving stress and burdens

Spans the financial, emotional, and physical stressors pertinent to the unique strains of caring for a seriously ill child. Participants often shared that parents of well children cannot empathize with the toll of medical parent-caregiving, which *providing options for respite care* may relieve. *Avoiding child's unplanned and non-critical hospitalization* reduces the stressors involved with hospitalization, and the inconveniences associated with looking after the child away from home; similarly, providing *home visits to provide medical treatment or care* reduces stress associated with seeking hospital care, exemplified in the following:

“I always [weigh] my options to see whether it is crucial for him to go to the hospital or just stay at home and get the homecare nurses to tend to him […] it helps me a lot because the process of him being in the hospital is always very stressful. […] Times when we have to bring [my child] into the hospital I always break down, because I just cannot deal […] So I try to avoid [bringing him in].”—PID16.

To ameliorate the operational and financial strains parents face, offering information on specialized transport for children with mobility challenges, guidance to available resources to reduce financial burden, and providing practical suggestions on reducing financial burden all can mitigate the demands of caring for seriously ill children, for example:

“[Our HCW team], they are sensitive in telling [us] not to buy things unnecessarily. They will help [us] to save costs, because it’s a journey, which costs a lot money […] While the hospital they will be offering you a lot of services. A lot of services, but a lot of money.”—PID28.

### 3.3. Theme 3. Collaborative and holistic care

This theme describes a shared journey where parents and HCW cooperate to maximize the child's emotional, physical, and psychosocial wellbeing.

#### 3.3.1. Shared decision-making

Balances between offering complete information on all management options for parents to make informed decisions while also supporting parents' preferences for involvement in decision-making. However, individual preferences for decision-making must be established early by HCW. For example, while most parents wished to be actively involved in treatment decisions, a subgroup of parents preferred to be medically guided. The contrasting view of PID03 and PID14 illustrates this nuance:

“The consultants explained to us…what, from their assessment, her condition is and would be… they helped us make an informed decision […] arranging us to meet and talk with these specialists [because they know we] want to know what it entails and the risks, and the benefits etc.”—PID03.

“When [our current team] came on board, it was good that they took on that [decision-making] responsibility. So I wasn't fighting with my husband […] I just wanted to… just follow the doctor. And they will figure it out.”—PID14.

*Recognizing and conveying the benefits for and burdens of technology and procedures on the child* are often raised for life-sustaining interventions. Although this can be a difficult discussion, it is essential to understand the family's assessment of meaningful benefit. Meaningful collaboration also involves HCW *being receptive to parental input and experience for better care* of the child given parents' experience with medical caregiving. This receptivity must be based on mutual respect rather than a parent-provider power differential:

“[…] They [kept] saying that he has a problem and [I knew he was fine] […] [but they said] we know everything because you are not an expert, you are just a patient, listen to me.”—PID17.

Ultimately, processes of shared decision-making should culminate in HCW *treating the child while considering the family's goals and preferences*:

“[At the end], they will say [clicks tongue] yeah *lah* [expressive slang], you are the mummy, you know what’s comfortable. […] there is a common understanding. I think that’s important.”—PID25.

#### 3.3.2. Holistic approach to care for child

Prioritizes the child's quality-of-life. Sociocultural barriers to palliative and supportive care provision exist at both parent and provider fronts. However, for all seriously ill children, *incorporating palliative and supportive care elements into clinical management* is often beneficial and appreciated when done in a sensitive manner and at an appropriate juncture. A subset of parents further discussed the value of *recommending comfort care in clinical situations where child's prognosis is assessed to be poor*:

“If you put a [tracheostomy] on, then he will live. Then [what]? So he’ll become a vegetable? […] He's on the bed, 24/7 […] my question to the health care providers will be… to what and what [for do] you want to continue that?”—PID25.

Given that HCW often care for these children long-term and across various care settings, parents value HCW who make efforts to foster a personal relationship with the child, create a child-friendly atmosphere in hospital, and provide emotional support and encouragement to the child, all of which establish a nurturing and comforting patient-provider relationship:

“They really show him care and concern […] it’s something that they don’t have to do… going above and beyond the call of duty, it’s actually more trouble for them. They could be just resting or like doing something else instead of having to bring him out for a walk.”—PID27.

Finally, to expand the child's identity beyond that of a sick patient, parents value *provision of facilities or services for child's play, engagement and involvement in school*. Because parents prioritize supporting their child's developmental and experiential growth, they actively seek services and interventions that foster ongoing neurodevelopment. This includes *facilitating access to inclusive schools for children with special needs* and *providing allied health care support to meet parents' goals for the child*. In this context, allied health refers to the group of non-physician medical professionals who possess specialized training and licensure, playing supportive roles in healthcare. This category encompasses various occupations, such as medical technology, physical therapy, social work, and more. To establish a shared understanding and ensure effective collaboration, it is crucial for the care team and parents to engage in open discussions and reach a consensus regarding these goals. This allows for a unified approach to care that addresses the aspirations of the parents:

 “To me, PT [Physical therapy] is quite important for a kid like her […] We often only meet the PT only in hospital. Then the PT in school [does not] really understand her and provides very little support for her […] we also very overwhelmed […] in the hospital [we have] such limited time [to] do everything […] we [cannot] absorb at that very short period of time […] [I just wish] they can visit [my child to do PT at home].”—PID19.

### 3.4. Theme 4. Efficient healthcare structures and standards

Parents highlighted how efficient healthcare structures and standards are fundamental in enabling HCW to deliver effective family-centered care.

#### 3.4.1. Accessible medical care

Emphasizes access to medical services to ensure that the child's complex needs are met. Facilitating access to multidisciplinary expertise in their child's range of conditions, availability of on-demand advice, and approachability for parents to seek advice from HCWs during medical emergencies are critical to lower barriers to care access:

 “[Our previous doctor] took his own initiative to be the main contact. Our contact. […] He arranged [all the various specialists] to see our baby [and] he’s so nice that he created a chatgroup. He said that after discharge, if anything, just give him a call [and for] anything urgent we can just message him.”—PID28.

*Providing convenient processes to obtain medical equipment and supplies*, and *assistance in acquiring high-cost medical equipment* are also critical in helping parents cope with the logistical demands of caring for a seriously ill child. Finally, *provision of sufficient financial support based on an assessment of family's needs* reflects the nuances of adequate self-perceived, rather than absolute, financial support:

“A lot more thought needs to be put into providing funding for the special needs [community] […] it’s very hard, it’s very sad know that at times, we need to [choose]. My child needs three items, and it’s mandatory, but I can only afford to buy one of it. So what do we do with the other two? […] I really urge you to [modify] regulations for fundings, because this is very, very important. we feel like we are being penalized for having a special needs kid […] we are just above the bottom line of the income cap… and we are literally this sandwich group.”—PID20.

#### 3.4.2. Effective administration and facilities

Reflect flexibility and efficiency in services. Attending to the child without undue delay at the Emergency Department and taking appropriate action to reduce child's exposure to other communicable diseases in healthcare facilities are regarded as core services. Parents also appreciate flexibility in administrative procedures and protocols to accommodate both child and parental needs, including providing flexibility for parents to choose their HCW, and allowing flexibility in number of caregivers for child during hospital admissions, as these are high-stress scenarios in which caregivers may feel overwhelmed. Finally, providing parents with a place to be close to their child in healthcare facilities is particularly salient for parents whose children are repeatedly hospitalized:

“[When] your child is in the hospital […] we should always be here just in case anything happens […] but the facilities in the ICU really cannot make it. […] you [can’t] stop work[ing]. And ours is long term […], you end up having back aches, neck aches, then you cannot last.”—PID34.

#### 3.4.3. Coordination and continuity of care

Reflects ways to harmonize care across HCW and institutions. *Alignment of care and management goals across HCW* is crucial to assure parents of their care team's cohesiveness in caring for the child, rather than the following:

“[Our primary consultant had not] agreed with it. But the team… wanted to do the surgery […] his heart wasn’t in it anymore […] he just had to go with the team. And [it made me feel like] he had just given up on [treating my child].’—PID14.

Furthermore, for a more seamless care experience, communication to ensure coordination across HCW and ensuring smooth transition of care across service delivery settings are essential:

“[When moving from one team to another within the hospital], the culture is very different and people are different and we need to pick up, we need to pick up that communication again […] get used to the management style of the case, which is quite different […] the treatment direction [was not] consistent throughout, instead [it was] changing and changing along the way, and it creates quite a lot of frustration and moments like, Hey, I thought we fought for it, and then we listened to you, and then only to find that it’s being reversed.”—PID02.

To reduce care fragmentation and ensure that their child's complex conditions are well-managed across wide-ranging specialties, parents prioritize having *a main HCW/team who consistently oversees child's medical needs*, and *a HCW/team who coordinates child's care between different disciplines.* Whilst these two entities may or may not be the same person or team, these roles ensure well-coordinated care for seriously ill children, including *coordinating appointments to reduce hospital visits:*

“…we were struggling [because] there wasn’t like a so-called primary doctor […] [my child] sees a lot of doctors and usually they just focus on their specialty […] but having one overall doctor in charge, who really understands the case and understands the family needs [would have helped us a lot].”—PID07.

## 4. Discussion

Our study developed key process indicators that are important to parents of seriously ill young children across various service delivery settings and throughout illness trajectories. We also examined how these care processes collectively contribute to quality of care in an overarching framework from the parental perspective. Being responsive to parents' priorities not only directly impacts the well-being of the child (29), but is also associated with better outcomes for child/parent dyads (30).

The PRICELESS framework has the potential to guide comprehensive assessment of quality of care and inform quality improvement initiatives for seriously ill children. Firstly, it highlights the importance of addressing components at both the outer ring and inner circle. Prioritizing parental and child access to and navigation of the care network is crucial for effective quality improvement, as these ensure services and care delivery reach end-users. Our findings differ from the traditional perspective of the Iron Triangle of healthcare (30), as we observed that parents do not perceive quality, access, and cost as three competing domains in healthcare. Instead, they view their child's illness as a continuous journey where access and ability to navigate the care system are fundamental to their evaluation of the care experience. Our study also identifies specific process indicators related to costs, embedded within the subtheme of “reducing caregiving stressors and burdens”, which deviates from the domain separation of the Iron Triangle. These findings therefore offer more person-centered insights for stakeholders seeking to maximize family-centered care.

Donabedian emphasized the crucial role of “process” in healthcare quality—ensuring effective and efficient execution of activities and interventions to achieve desired care outcomes. Thus, knowing what care processes to evaluate and enhance will directly impact child and parental outcomes and experiences. Without process measures, it is challenging to pinpoint specific areas for intervention or assess the effectiveness of care delivery (31). We also expand upon the findings of Kokorelias et al.'s scoping review, addressing the need for strategies that can be practically implemented across age groups for young children, illnesses, and care settings (23). Hence, the process indicators can be used to identify instances where priority services for young children with serious illnesses from birth through 8 years may be underperforming and thus should be the focus of future efforts.

To our knowledge, the process indicators and resulting PRICELESS framework are the first to potentially apply to a wide spectrum of seriously ill children and diverse service providers and encompasses a broad range of healthcare settings, including non-clinical services like community-based therapy. We also adopted an inclusive definition of HCW to include providers from various disciplines and thereby creating a broader appeal across the care continuum. Importantly, we found that parents do not necessarily separate quality of care priorities based on disciplines or settings, but view individual healthcare workers as integral parts of their child's care network. Similarly, we learned that parents may not explicitly distinguish between the responsibility of the healthcare system vs. social care like special schools and community agencies.

The concept of interconnectedness of HCW within a child's network of services, which is represented in our framework as the inner circle, highlights the interdependence of HCW in delivering comprehensive care. Parents value the “collaborative and holistic” nature of care, appreciating the contributions of each HCW within the larger context of their child's healthcare journey. By acknowledging the integrated nature of the care team, healthcare systems can foster a more cohesive and family-centered approach to providing care for children. These findings provide impetus for cooperation between health and social care, and toward synergistic partnerships that overcome traditional silos of fragmented care. Indeed, calls for greater integration and coordination of care have been a dominant theme in recent years (32–34). This may be even more pertinent for seriously ill children and their families who frequently have complex health and social care needs (34–36).We also learned parents conceptualize the various providers involved in their child's care as part of the “family”; these providers have the potential to play pivotal roles in “supporting parent-caregivers” and “reducing caregiving stressors and burdens”. This partnership is reminiscent of a family-centered medical home model which prioritizes accessible, comprehensive, and enduring care in the context of family and community (32, 34). Parents often referred to healthcare providers as “our doctors”, particularly when a trusting relationship had been established, highlighting the importance of caring for the parent/child dyad within the framework of family-centered care. Viewing these dyads as care units also emphasizes the importance of being responsive to parents' needs in addition to those of the child. It also establishes a healthy patient-provider relationship that supports the whole family throughout their care journey.

### 4.1. Strengths and limitations

This study's strength lies in its robust methodology. It included a broad range of parental perspectives across diverse service settings and serious illness categories. We explored the perspectives of a unique population of parental caregivers who, on top of “typical parenting”, assume an intricate combination of roles extending across physical, emotional, social, and spiritual domains (29). Responsibilities often include being a care provider, medical and financial decision-maker, patient advocate, care coordinator, advocate in education, communicator, transport service provider, and income-earner—all in one (37). For these parents, the child with serious illness(es) has complex needs that are not stratified along specific diagnoses or types of specialist care. Our findings substantiate the importance of being part of their unpredictable journeys, recognizing multiple roles that families of seriously ill children undertake, and revealing many opportunities (and processes) to better support them. Further, our study lends weight to the importance of ensuring coordination and continuity in care in health systems that have been historically fragmented (38).

Aspects of these findings, though meaningful, may not be transferable to other settings. For example, where out-of-pocket costs are lower, such as England's universal healthcare system where healthcare is publicly funded and free at the point of delivery, financial priorities in PRICELESS may not be relevant to parents. Results may also not be applicable to acute care setting, whose conditions are less likely to require longer term care, or to lower income countries with service access issues or structural gaps in the healthcare system. Furthermore, in grounded theory research, the interpretation and subjective analysis of data play a significant role, which can introduce bias and potentially influence the findings. Despite our efforts to minimize these biases, it is important to acknowledge that qualitative research inherently involves a higher level of subjectivity compared to quantitative data. Our study only captured the perspectives of specific ethnic and religious groups in Singapore. Therefore, the findings may not fully applicable to other populations. By explicitly stating our objectives before conducting interviews, it is possible that parents might have modified their responses to advocate for specific services or provided socially desirable answers, instead of sharing their authentic thoughts or perspectives. Finally, we acknowledge that the processes of care we have identified in the PRICELESS framework may not apply to the equally important journey that bereaved parents or children of older ages make.

### 4.2. Conclusions

The 64 process indicators generated in this study can be used to develop parent-reported experience measures of quality of care for seriously ill children. This will enable standardized measurement and service benchmarking (39) for a vulnerable population in which process assessment needs further exploration. We posit that the components of the PRICELESS framework can pragmatically guide the design and delivery of quality initiatives. Combining the process indicators and framework components offers opportunities for implementing and evaluating multi-component interventions to improve quality-of-care for seriously ill children. As one parent concluded: “In just listening to the voice of the mom or the dad… you’re actually giving [us] a chance to speak up… and ask yourself [what you need to improve]”—PID06.

## Data Availability

The raw data supporting the conclusions of this article will be made available by the authors, without undue reservation.
