# Supplementary material for: A qualitative exploration of parental perspectives on quality of care for children with serious illnesses
Source: Front Pediatr. 2023 Jul 28;11:1167757. doi: 10.3389/fped.2023.1167757 (PMC10419205; doi:10.3389/fped.2023.1167757)
Supplement: Supplementary file 2 [file Datasheet2.docx]

# Supplementary File S2. Interview Guide

# A Qualitative exploration of Parental Perspectives on Quality of Care for Children with Serious Illnesses

## INTRODUCTION TO PARENTS

In this study, we will explore what are important and relevant processes of care to parents of children living with a serious illness.

We aim to develop the first grounded theory framework of care services and behaviors deemed important and relevant to parents of children living with a serious illness. Eligible parents will be recruited from multiple centers, hospitals and organizations. Following an informed consent, a one-to-one in-depth interview will be arranged at a place (either in-person, phone, or videoconference), date and time of his/her preference, and complying to all prevailing Covid-19 regulations.

## Maintaining professionalism in clinical research

Researchers shall uphold the profession’s responsibility to society by promoting ethical and professional practice standards, and to operate within the scope of practice outlined within those professional guidelines. Researchers shall respect and safeguard the welfare and rights of all individuals with whom they interact professionally, including but not limited to research participants. Researchers and interviewers should treat their patients, their families and members of the public with utmost respect and dignity. They have a duty to society as a whole, and must be conscious of their place in society and the research arena to ensure that all aspects of the privacy of research participants and their families is respected prior to, during, and following, any research project.

## In-depth, narrative interview process

The interview format is intended to be narrative and conversational. While there is a semi-structured interview guide, the interview should flow naturally. We will focus on allowing the participant to raise topics and speak in-depth about their experiences. We will allow the participant to take the lead and allow the topic to evolve naturally. We will continue to the next question if the prior topic has been completed, or if the participant no longer wishes to speak about the topic.

# Interview guide for parents

[Intro]

Hello _________, my name is ____________. Firstly, I want to thank you for being here and agreeing to share your experience.

We are trying to understand **what** and how to ensure the **care** delivered by (child’s name’s) health care providers is **high quality care** from parent’s perspectives.

What we want to identify is what parts of **care is important** to (child’s name) and your family. Another way to explain it is that we want to **clarify what services and behaviors** are important to you from his/her health care providers.

At the end of the study, we aim to **improve families’ care journeys** by measuring how well health care providers are delivering care for (child’s name) and your family, a bit like quality assessment.

Before we begin, as outlined in the consent, everything you share will be anonymous. I will not use any specific names or places in reporting what I learn from these interviews or identify you or anyone you mention in any way. Remember that there are no right or wrong answers.

I will also be recording the interview; but, I will not share this recording with anyone outside this study. I also want to emphasize that you may stop at any time and for any reason during the interview.

***Do you have any questions?***

Ok! Let’s get started {turn recorder on}

***Open with non-sensitive topic: warming up to the family***

[I would like to get to know your family and (child’s name) a little better.]

| Can you tell me a little bit about yourself? Can you tell me a little bit about (child’s name)? |
| --- |

***Exploring parent’s perception of important processes over the trajectory***

[I understand that (child’s name) is now living with (diagnosis, if definitive) OR (complex medical condition/s).]

| Can we start at the beginning—**how** did you come to know that (child) was ill?   - *Probe: Who informed you, and how?* - *Probe: How did you/your family respond to the news?*   Was there anything (child’s) health care providers did that was **important** when you were in the process of accepting the diagnosis?   - *Prompts: could you be more specific?/could you tell me more about that?*   Was these anything (child’s) health care providers did that was important to you which you would have preferred to be done **differently**?   - *Prompts: could you be more specific?/could you tell me more about that?*   [Added to interview guide at later stages] If you don’t mind, I would like to explore how these ideas may be related.   - *Prompts: Are ____ and ____ supporting or intersecting? Distinct? Dependent?* - *Probe: Were there foundational elements that really stuck out?* |
| --- |
| Can you tell me what a typical day is like for (child’s name)?  [This question is intended to elicit child’s perceived needs and potential gaps, without being too straightforward]  Based on what you just shared, does (child’s name) face any **struggles** that **affect his/her** daily routine?   - *Prompts: barriers are like obstacles or hurdles, things that stand in your way* - *Can you be more specific?*   Based on what you just shared, what is important to you that **helps you** with his/her daily routine?   - *Prompts: facilitators are like aids, things that make things easier for you/do you have any examples?)*   What do you find, for (child’s name), **are the important things** being done by the health care providers?   - These may be **services and behaviors** that help (child’s name) with his/her day to day or care? - *Prompts: could you share why you think so?/can you share a little more?*   Are these services and behaviors **being delivered** by (child’s name) health care providers?   - If yes   - Could you share a specific example of the services or behaviors? - If no   - Could you share what you think are the current limitations/ challenges as to why these services or behaviors are not delivered?   - Could you also share what is needed or how things should change, so as to ensure these services or behaviors are delivered? |

[Reaffirm appreciation for the parent having shared such sensitive or personal information.]

***Exploring parents’ expectations and needs***

[I would also like to understand what is important and relevant to you as (child’s name) mother/father.]

| How is a typical day like for you?  [This question is intended to elicit parent’s perceived priorities and potential gaps, without being too straightforward]  Based on what you just shared, do you face any struggles that **affect your** daily routine?   - *Prompts: barriers are like obstacles or hurdles, things that stand in your way/can you be more specific?*   Based on what you just shared, what can or do health care providers do which **helps you** with your daily routine?   - *Prompts: facilitators are like aids, things that make things easier for you/do you have any examples?)*   As (child’s name) parent, what are the **services or behaviors you feel are important** from (child’s name) health care providers?   - *Prompts: These can be medical or non-medical/can you tell me more?*   So based upon what you just described with your own struggles, your own needs, what do you prioritize for healthcare providers to do in terms of services or behaviors that actually meet these needs?   - *Prompts: could you share why you think so?/can you share a little more?*   [Added to interview guide at later stages] If you don’t mind, I would like to explore how these ideas may be related.   - *Prompts: Are ____ and ____ supporting or intersecting? Distinct? Dependent?* - *Probe: Were there foundational elements that really stuck out?* |
| --- |

[Reaffirm appreciation for the parent having shared such sensitive or personal information.]

***Exploring improvements to current care***

[Lastly, we want to explore other ways that current care can be improved to better meet the needs of families like yours.]

| Based on your experience with various providers in (child’s name) care network, what services and behaviors **have been beneficial** for (child’s name) and your family?  What services or behaviors do you think **can be improved** for (child’s name) and your family?  What do you think is important to ensure that high quality care is provided to (child’s name) and your family **in the long run**?   - Are there any resources or services that are important?   If we have not covered it, what else do you think is **important or relevant** for you or your child, that will improve your family’s care journey? |
| --- |

We have come to the end of our discussion. You have provided us with a lot of insight into the aspects of care that are important to you. We will do our best to translate what we learn into a meaningfully improved experience for families and have plans for follow-up studies to measure the quality-of-care. Please rest assured that everything you say is confidential and none of the topics mentioned will be attributed to any one person. I hope you had a good experience.

[Thank the participant for his/her time and honesty. Offer to answer any questions.]
