# Supplementary material for: A qualitative exploration of parental perspectives on quality of care for children with serious illnesses
Source: Front Pediatr. 2023 Jul 28;11:1167757. doi: 10.3389/fped.2023.1167757 (PMC10419205; doi:10.3389/fped.2023.1167757)
Supplement: Supplementary file 3 [file Datasheet3.pdf]

Supplementary File S3. Summary of 64 process indicators (specific, well-defined care processes describing how particular practices, interactions, services, or procedures take place) and respective illustrative quotes constituting quality of care from the perspective of parents of seriously ill children			
**Theme**	**Sub-theme**	**Process Assessment indicator**	**Illustrative quote(s)**
**Theme 1. Professional Qualities of HCWs**	**Responsive and sensitive communication (9 indicators)**	*1. Healthcare workers present themselves in an honest manner*	"[Our first surgeon] was soooo [scoffs] so terrible [scoffs again]. He said to me that he wouldn't have done the surgery… that it was a mistake […] [After he conducted the surgery], she actually ended up with a chest infection… And [my husband reported it to] the doctor […] I think [the doctor] knew... I think it was his chance of like… you know, trying to… let her go … because if [my husband] didn't [report the lump], she would have died."—PID14<br>"Most of them that I've met have been so nice and giving good advice, starting with the private PD that I have. When it hit us in the private hospital in [the private hospital] right. So, I I think he is very ethical in the sense [that] they can [keep] [sic] us in the private hospital, long term, clearly, from the income point of view we could be their cash cow. Yeah, but they sent us over, they said they feel that this this is not easy to handle, go to a structured hospital at least we get government subsidies because this could be a long term process. I think they are very kind to us and subsequently for the next half year even though we are in [the tertiary referral hospital] they have gave us calls to follow up as well on the progress and things like that so I think they have been very nice."—PID15
		2. Making efforts to build parental trust in healthcare workers	"[After our whole journey], we literally trusted [our doctor] a lot… and we always have a discussion. Yeah. So we felt that he really really very sincere. And yeah, and we are very thankful that we met him *lah* [slang]. "—PID28<br>"I mean [the healthcare team and I] are friends until now they will come and see [my child] off schedule. And so those things are the things that [showed me] they are very transparent. They will say… Don't buy it here [because it's more expensive in this hospital] […] go to this shop [somewhere else] to buy it."—PID25.
		3. Respecting the parental right to information	"Firstly, priority, regardless of what you're doing you need to communicate it to me, so that I know what's going on with baby *lah* [slang], medical or non-medical. [...] the doctors and the nurses there, should know, will know, and they should let me know." —PID31
		4. Providing information on child's condition in a timely manner	"They [did] not really update us about what [was] going on. […] On the week of discharge then we realized [wow], actually [my child has] so many [issues]? […] I don't know what I don't know! […] Now too late already then you tell me [sic]?" –PID26.
		5. Communicating in a manner that is sensitive to parent's needs'	"I have experience with doctors that are very blunt, very straightforward, very scientific and diagnosis [sic] that can tell you that 'Oh, your child probably cannot walk um, you know, for the rest of your life'… at that point of time it really breaks a, a mo- a first time mom's heart. [voice breaks] [...] most of them need to be [scoffs] sent for communication lesson. […] for a mum who is trying to accept the reality of their child being not typical, it's very very very hard for them to hear this truth. Even though it's facts." —PID20
		6. Using understandable language and methods to communicate	"They helped me understand by speaking in a simple language instead of a 'Doctor' language." –PID10<br>"He explained very patiently and thoroughly and he will draw every time. Every time, every time he, every time we discuss something he will draw. Yeah, he will have paper and pen with him. Yeah! [...] that plays a very, it really helps us *lah* [slang] in understanding [my child's] condition inside" —PID18

		7. Giving parents time and space to make decisions without pressurizing them	"They somehow like to push you to do, to decide on things, when things can be taken at a slower pace... they like to push people to go home fast […] they should [not] push parents [who aren't ready]..." —PID06<br>"[…] before um, they discharged us from the hospital […] they helped us to make that decision. They gave us a lot of time." —PID02.
		8. Respecting the spiritual or religious customs and beliefs of parents	"Spiritual support was, was key for us going through the tough period. […] even though we were emotionally and mentally drained… we managed to [find] peace and get through that very, very difficult period." —PID17.
		9. Avoid causing additional stress due to a lack of appropriate sense of urgency when communicating with parents	"The doctor [woke] me up at around 2am. I thought that something happened to my child. And the only question that she want[ed] to ask? Whether [any] of your family members smok[e]!" —PID20
	**Competency of healthcare delivery (6 indicators)**	*1. Ability to identify and treat child's medical issues*	"We are quite grateful that it was picked up very, very early on and then early intervention can just start ASAP [as soon as possible] […] they were, they were doing *everything*." —PID33
		2. Delivery of timely medical care	"[HCWs would] prioritize [my child's] issues [and] come by very quickly […] and have a look and just really check her to make sure that generally she seems stable." —PID07.<br>"[…] they won't just brush it off like that, they will ask the doctor, like this specialist to come and take a look, make sure… I can be assured that he is in good hands." —PID27.
		3. Providing symptom management to ensure child's physical comfort	"There was one period where he couldn't sleep at night [...] they actually make amendments to his feeding hours. [...] after the amendments, right, he does sleep better!" —PID28
		4. Avoiding unnecessary treatments and investigations on child	"The nutritionist will want to up her feed until we can leave… It *has* to be a certain rate… they just want to do all this stuff [...] they will want to put a drip in her. And she has very bad veins. So… they're basically popping, they're basically, trying to find a new vein every day.... it's quite traumatic for her." —PID14.
		5. Reassuring parents of healthcare worker's expertise in the field	"At the moment of time, [he assured us to] follow him. Nothing [would] go wrong if we follow him because he gave us the assurance that he knows what he's doing. And he's keeping track of uh [my child's] results." —PID32.
		6. Taking responsibility and being accountable for child's wellbeing	"I started staying very long hours with her after [my child was injured under their care] […] until now there wasn't a concrete or an acceptable conclusion to this [incident] […] And my trust level went down to zero for that." —PID31.<br>"[…] I was chasing one of the medical social worker for like two weeks […] no response from them. No response, you know? No response via call, no response via uh, emails..." —PID21
**Theme 2. Supporting parent caregivers**	**Empowering parent-caregivers (6 indicators)**	*1. Equipping parents with skills to confidently deliver out-of-hospital care*	"[Our healthcare worker] walked us though, equipped us with medical knowledge […] You know what to calculate what to look for, how to handle, not to panic, etc. So we are uh, taught to do so […]"—PID15.
		2. Providing anticipatory medical advice for parents to recognize when child's condition deteriorates	"She taught me from A to Z. What to look out for, how to actually determine whether this is serious, and you need to bring him to the hospital."—PID12.
		3. Acknowledging and affirming parents' efforts in caring for their child	"They always like encouraged us like […] You're good! You're amazing! […] Even though we do something like something we think is our fault. They say 'No, no! it's not your fault!' […] really one of the highlights and I think that's [kept] us going. " —PID18.

		4. Providing parents with opportunities to bond with children during admissions to healthcare facilities	"I just want to create this bond with him. Like, you know, I did not get like, skin to skin… didn't have that with him. [...] I was just sitting there, not doing anything, just [sitting] there staring at him."--PID35
		5. Opportunities for caregivers to advocate/speak up for their child	"[…] you can always disagree, but […] give the caregivers a chance to voice out for their child. Especially like my child, she is nonverbal […] I am her voice. If I don't tell you that she deserves this, then who else is she, who else can she rely on?"—PID06.
		6. Providing parents with opportunities to give back to the special needs community	"This is the experience that I can share… to mothers who feel that, you know, I'm already at my wit's end […] so things related to studies... I will always tell myself like, Okay, why not? We try. It might not necessarily help me. But... it might help other parents."—PID16.
	**Providing psychosocial support to parents and family (7 indicators)**	*1. Showing parents genuine care and sincerity*	"They are really part our journey. It's not like 'oh, you're just a normal patient' kind of thing. They really, like, get to know us well [...] form friendships with the parents… I'm so close to them, I can just talk to them one to one, midnight chat." —PID32
		2. Supporting parents' hopes for their child	"I was heartbroken... especially because the doctors painted a picture of a future [that] is really, really bad... hearing that is actually very scary... you are pushed to the corner where you have no other choice... You [just want] some hope that, you know, termination is not the only choice. I would have liked to speak to someone else." —PID07<br>"That day was…we already lost ourselves. We [broke] down and then the doctor [kindly said], ok, we'll take things slowly. It won't be bad, it will be okay."—PID18
		3. Preparing parents for what may lie ahead	"He gave us a what if this, what if that [...] to see our journey in a, in a bigger picture […] I don't want to just be in cloud nine, but you know, someone needs to pull me to the ground."—PID35.
		4. Providing parents with a compassionate listening ear	"That moment was very important to me. Like being able to at least talk to somebody […] me, being the adult, I am also very lost. I don't know how to, like keep myself calm and composed […] finally, somebody whom I can talk to and let my guard down. I really cried in front of her *lah* [slang], like, because I needed the emotion let out."—PID34.
		5. Providing parents with emotional/physical space to grieve	"Space in the sense that I need to…. I need to grieve [...] there's just too many people coming in... can't you see me? [...] I was not in a proper state of mind to actually hold a conversation. [...] I felt that my privacy was invaded somehow." —PID35
		6. Accessibility to parent support networks	"Being in that group, support group, was very useful […] we have parents from different hospitals or even backgrounds coming together… being able to come together and share [was] really useful."—PID03.<br>"There was no support group to fall back on. It was, it was a struggle really… to face everything alone […] if you allow these parents and other parents who are facing similar conditions, you bring them together, it actually helps a lot."—PID06.
		7. Attending to the psychosocial needs of the family unit resulting from child's condition	"[My child's] other three siblings are emotionally supported as well. Because it's also a hard time for them […] [the team does their best to] ensure that… their emotions are well taken care of so that their studies won't be affected."—PID12.
	**Reducing caregiving**	*1. Providing options for respite care*	"Because being the main caregiver is not easy [...] I'm also working […] finally, with this [respite care option], we are able to actually have self-care […] if not I was always on the edge, was always angry, you know? Tired. […] People didn't understand… Why is it so important for you to rest? Is it [because] you don't love your child? […] [They didn't understand that] I need to be strong, I need to be… sane, to take care of my child!"—PID13.

		2. Avoiding child's unplanned and non-critical hospitalization	"I always [weigh] my options to see whether it is crucial for him to go to the hospital or just stay at home and get the homecare nurses to tend to him […] it helps me a lot because the process of him being in the hospital is always very stressful. […] I try to reduce the stress level [by avoiding going to the hospital] [...] times where we have to bring into the hospital I always break down, because I just cannot deal."—PID16.
		3. Home visits to provide medical treatment or care	"They will try and you know, and prioritize her issues [and] come by very quickly […] really check her to make sure that generally she seems stable and if she's really not doing well, then they will tell us you need to go to the hospital"—PID07.
		4. Guidance to available resources to reduce financial burden	"For the financial, they support... 100%. [For any] hospital admission or her equipment that she needs, then they will support, try to find all the funding for her [...] try their best to get whatever she need." —PID08
		5. Provision of practical suggestions on reducing financial burden	"[Our HCW team], they are… sensitive in telling [us] not to buy things unnecessarily. They will help [us] to save costs, because it's a journey, which costs a lot money [...] While the hospital they will be offering [us] a lot of services. A lot of services, but a lot of money."—PID28.
		6. Offer information on specialized transport for children with mobility challenges	"They sent a driver to come and fetch [my child] to go their center to actually take this art therapy thingy you know, storytelling [...] I cannot send him but I would love him to attend so that he won't be bored at home. So they say I can send someone to fetch [my child] to and fro! " —PID12
**Theme 3. Collaborative and holistic care**	**Shared decision-making (5 indicators)**	*1. Offering complete information on all management options for parents to make informed decisions*	"The consultants… explained to us what [the options] entails and what, from their assessment, her condition is and would be… which um aided us… helped us make an informed decision […] arranging us to meet and talk with these specialists […] being parents of course… having to make decisions like this, that will impact [your child's life], you want to know what it entails and the risks, and the benefits etc."—PID03.<br>"I wish that, you know, we could have [been given] another option. […] an option to speak to someone else, you know, that, besides termination, knowing that there are resources and there are support [...] rather than just thinking, Okay, it's either termination or I just choose to keep, and then [if] I choose to keep, I'll be all alone. I was kind of really feeling that I was all alone." —PID07
		2. Supporting parents' preferences for involvement in decision-making	"When [our current team] came on board, it was good that they took on that [decision-making] responsibility. So I wasn't fighting with my husband [...] I just wanted to… just follow the doctor. And they will figure it out." —PID14
		3. Being receptive to parental input and experience for better care	"If they are willing to listen to the caregiver and willing to work together, they themselves can learn from caregivers […] you just shut the door which could have been open." –PID06.<br>"[…] they kept saying that he has a problem [but I knew he was fine] […] [but they said] we know everything because you are not an expert, you are just a patient, listen to me. That kind of thing."—PID17.
		4. Recognizing and conveying the benefits and burdens of technology and procedures on the child	"If the equipment can help her survive...become normal or [something], we will, by all means, spend the money. […] but [clicks tongue] she's like suffering every day… waiting [for her] time to [go]… meaningless that she keeps lying down there and she can't do anything also." —PID19
		5. Treating the child while considering the family's goals and preferences	"[Under the previous team] I had to keep on advocating for her […] they [didn't] really understand why [I wanted things this way] [...] And I have to explain myself. And it's very wearisome... we don't want that for her. [...] [when our current care team came on board], they, they sat us down and they wanted to hear our story. And they said, Okay, this is what we're going to do [...] it became a lot easier to advocate for [my child] to say no, no, no." —PID14
	**Holistic approa**	*1. Incorporating palliative and supportive care elements into clinical management*	"I know [the palliative doctor's] intention was to really support us and to provide the best care but at that point [...] it's very untimely. I'm very scared, the moment I see her, I will feel that, you know, that's the end of the journey for my son." —PID32.

			“The doctor needs to say I think you should take palliative. [sniffs, voice breaks, crying] It's the hardest thing for a mother to say I want palliative. [stuttering, voice is uneven, strained] […] here, it's just advocated to do abortion, um, [whereas] palliative care for a child… it's not really looked on.” —PID14
		2. Recommending comfort care in clinical situations where child's prognosis is assessed to be poor	“If you put a [tracheostomy] on then he will live. Then [what]? So he'll become a vegetable? […] He's on the bed, 24/7 […] my question to the health care providers will be… to what [end] and what [for do] you want to continue that?”—PID25.
		3. Make efforts to foster a personal relationship with the child	"They really show him care and concern [...] it's something that they don't have to do… going above and beyond the call of duty, it's actually more trouble for them. They could be just resting or like doing something else instead of having to bring him out for a walk.” —PID27
		4. Creating a child-friendly atmosphere in hospital	"They know that we cannot really get outdoors. So they try to like have events in the clinics, or you know, they [create a space] where they will carry out events, then they try to plan things that they can do within the hospital space, which is much safer for [the children].” —PID35
		5. Providing emotional support and encouragement to the child	“I can see from my child here her mental [state], she's more traumatized [...] I'm afraid [she] will lose hope of living […] we need to tell them that even though you are sick, you still can do It… they need motivation.” –PID13
		6. Provision of facilities or services for child's play and engagement	"The social aspect of the [early intervention] program would be the most valuable part of it... having other friends-so that she can watch and learn and play with and imitate, and also the teachers, the teaching aides and the teaching staff in the class is something quite valuable in the end.” —PID03
		7. Facilitating access to inclusive schools for children with special needs	"I asked [an early intervention centre and the healthcare workers] if there any other schools that you're working with? That are willing to accept special needs? So they opened up the door for [our current kindergarten], which we're very thankful [for].” —PID25
		8. Providing allied health support to meet parent's goals for the child	"To me, PT [Physical therapy] is quite important for a kid like her [...] We often only meet the PT in, only in hospital. Then the PT in school… don't really understand her and provide very little support for her. [...] we also very overwhelmed [...] in the hospital [we have] such limited time [to] do everything […] we [cannot] absorb at that very short period of time [...] [I wish] they can visit like, to see [BY]'s entire condition and then just [have] one hour, two hour sessions to do all this physio [...] we need to do at least to do PT [so as not to] worsen her condition [...] so that's why um, constantly giving physio is necessary.” —PID19
**Theme 4. Efficient healthcare structures & standards**	**Accessible Medical Care (6 indicators)**	*1. Facilitating access to multi-disciplinary expertise in child's range of condition(s)*	"A whole suite of, of, of doctors just there, you know, when you need it [...] more than one brains thinking for your baby [...] thinking like, what is the issue? How they can solve the issue? So I can really tell that, you know, they were they were doing everything. Like they were cracking their brains to get it. ”—PID33
		2. Being approachable for parents to seek advice	“[…] everyone that we try to ask for some advice or help… are very helpful […] whenever we try to call them... They always help us, inform us, this is what you're going to do.”—PID01.<br>“She [gave] me this assurance that we are here for you... If you have any medical concerns that you think that you are not able to identify [by] yourself, I'm always here for you to ask any questions […] I [always] go to her first.”—PID12
		3. Availability of on-demand advice	"We've been keeping in very close contact [...] they will quite quickly reply [...] So it's a very, proactive communication, dialogue between, between us and the team [...] We can have, very instantaneous, I won't say instantaneous *lah* [slang]- rapid response.”—PID31

		4. Providing convenient processes to obtain medical equipment and supplies	"There's quite a fair bit of logistics involved caring for someone with medical condition... [...] its very challenging [...] on top of her daily care... there's this delta, this this difference that most parents don't have to manage, right, which is her medical supplies... It can like leave a, you know, be a significant part of their home care *lah* [slang] at home."--PID03
		5. Assistance in acquiring high-cost medical equipment (e.g., ventilator, suction machine, buggy)	"The hospital has loaned machines [...] we cannot use those normal like, off the, over the counter suction things for babies. So, they passed us a special suction that they use at the hospital [...] [our doctor] actually got the most senior nurse to approve all these loan machines for us."—PID33
		6. Provision of sufficient financial support based on an assessment of family's needs	"[…] feels like we are being penalized for having a special needs kid […] we are just above the bottom line of the income cap right, and then we are not able to get the subsidy. And then we are literally like this sandwich group you know."—PID20.
	**Effective administration and facilities (5 indicators)**	*1. Attending to the child without undue delay at children's emergency*	"Admitting to A&E is a big [sighs] is a big burden because the queues are long. [...] I don't want to waste my time. And I don't waste his time when he goes to go to school, because as he gets older, so I mean, I'm not trying to be difficult, but you need to understand from my perspective as well... "—PID25
		2. Taking appropriate action to reduce child's exposure to other communicable diseases in healthcare facilities	"This one is quite important for baby *lah* [slang], because the premature baby is quite weak, if [she catches] any bacterial infection then it's very bad for baby. I think [the hospital took into] consideration and good care for that. "—PID30
		3. Providing flexibility for parents to choose their HCWs	"The providers that we found eventually were, you know, the team that we built now, which is the same team that I've recommended to new parents… an amazing team. But [before we built this team] [...] the only good person I guess we found at the time, he couldn't help us. Because he was part of [another hospital] he had to refer us to the [other hospital's] [specialists], which was a nightmare." "—PID05
		4. Allowing flexibility in number of caregivers for child during hospital admissions	"I think they need to be more flexible with special needs child [in the hospital] […] the person in front [said], only one person can go in. Then I [said], but I need help!"—PID09.
		5. Providing parents with a place to be close to their child in healthcare facilities	"So, when he transferred to the Pediatric ICU, because the [temporary place of respite] is attached, they helped me to get a room there... I spent [many] months there... It helped me a lot. So I can at least work and attend to him […] For that we are very grateful. " --PID 02
	**Coordination and continuity of care (6 indicators)**	*1. Alignment of care and management goals across HCWs*	"[Our primary consultant had not] agreed with it. But the team… wanted to do the surgery […] his heart wasn't in it anymore […] he just had to go with the team. And [it made me feel like] he had just given up on [treating my child]."—PID14.
		2. Communication to ensure coordination across HCWs	"[…] Why do you all keep taking those notes so much? There's a reason right? So go and read it! Don't go and ask me! Especially when we are totally anxiety [sic] you know? […] this doctor asks the same question, this doctor asks the same question, this person same question."—PID21
		3. Ensuring smooth transition of care across service delivery settings	"[When moving from one team to another], the culture is very different and people are different and we need to pick up, we need to pick up that communication again [...] get used to the management style of the case, which is quite different [...] the treatment direction- it would have been good if it's consistent throughout instead of like changing and changing along the way, and it creates quite a lot of frustration and uh like, the kind of moment like, Hey, I thought, we fought for it, and then we listened to you, and then only to find that it's being reversed. "—PID02

		4. Coordinating appointments to reduce hospital visits	"[When all our appointments are put] together... it's not only more productive, it's also easier... to manage for the kid *lah* [slang]. If not, *wah* [slang] if I need to go and see the doctors three times [every month], then I think it's very challenging [...] it saves you the hurdle of having to make multiple trips"—PID11
		5. A main HCW /team which has consistent oversight over child's medical needs	"…we were struggling […] [because] there wasn't like a so-called primary doctor, that coordinates everything, because [my child] has so many issues. So she sees a lot of doctors and usually they just focus on their specialty […] having one overall doctor in charge, who really understands the case and kind of understand the family needs [would have helped us]."—PID07
		6. A HCW/team which coordinates child's care between different disciplines	"[...] we are grateful there's this liaison role... our central contact through, through all the various departments. And yeah, so that person has been most crucial [...] that is the point of contact that we feedback when we face problems, and where can we meet, who to meet because we just we don't have the contact of everyone. [...] I am relieved that someone [coordinates for us]- if not, I think we [would be] running [around] and it's very difficult to contact various departments. "—PID15
